# Neuronal activity triggers secretory autophagy to mediate the extracellular release of SNCA/α-synuclein

**DOI:** 10.1080/27694127.2024.2410683

**Published:** 2024-10-07

**Authors:** Yoshitsugu Nakamura, Shigeki Arawaka

**Affiliations:** Department of Internal Medicine IV, Division of Neurology, Osaka Medical and Pharmaceutical University Faculty of Medicine, Takatsuki, Osaka, Japan

**Keywords:** SNCA/α-synuclein, secretory autophagy, neuronal activity, extracellular vesicle

## Abstract

Autophagy has two distinct pathways, degradation and secretion. Autophagic degradation plays a pivotal role in cellular homeostasis by the formation of a double-membrane autophagosome in concert with numerous ATG (autophagy-related) proteins. However, the mechanism that mediates autophagic secretion is not fully understood. To explore how autophagic secretion is physiologically triggered and regulated in neurons, we investigated whether neuronal activity affected autophagic secretion by analyzing SNCA secretion in mouse primary cortical neurons and SH-SY5Y cells. In primary neurons, rapamycin promoted SNCA secretion, while the effect was canceled in primary neurons of *Becn1*^+/–^deficient mice. Stimulating neuronal activity by glutamate promoted SNCA secretion, autophagic flux, and colocalization of SNCA with LC3 (microtubule-associated proteins 1 light chain 3). These effects were inhibited by the intracellular Ca^2+^ chelator BAPTA-AM. Additionally, glutamate-induced SNCA secretion was suppressed by *Atg5* or *Rab8a* knockdown in SH-SY5Y cells, and mainly occurred in the fashion associated with extracellular vesicles in primary neurons. These results suggest that neuronal activity triggers autophagic secretion for releasing SNCA via an increase in intracellular Ca^2+^ concentration.

## Abbreviations

ATG: autophagy-related; LAMP1: lysosomal-associated membrane protein 1; LC3: microtubule-associated proteins 1 light chain 3; MTORC1: mechanistic target of rapamycin kinase complex 1; SOD1: superoxide dismutase 1

Autophagy has been investigated as a mechanism to degrade unwanted intracellular components, including proteins and organelles. Autophagic degradation plays an important role in cellular homeostasis against stress and diseases. In addition to the degradative pathway, autophagy is shown to have the diverse pathway for releasing the cargo extracellularly, which is referred to as secretory autophagy. However, how secretory autophagy is physiologically triggered and regulated is not fully understood.

SNCA/α-synuclein is one of the aggregate-prone proteins. The formation of SNCA aggregates in cells and the cell-to-cell spread of SNCA aggregates in the brain cause neuronal loss in synucleinopathies, including Parkinson disease, Lewy body dementia, and multiple system atrophy. Clarifying the mechanisms that maintain and disrupt SNCA proteostasis is key to understanding the pathogenesis of synucleinopathies. SNCA is degraded by chaperone-mediated autophagy and macroautophagy (hereafter referred to as autophagy). Autophagy is thought to be critical processes for SNCA proteostasis to prevent the formation of toxic aggregates.

Experiments using microdialysis detected endogenous SNCA in the interstitial fluid of mice. Additionally, neuronal activity induced by glutamate stimulated extracellular secretion of endogenous SNCA in primary cortical neurons, and neuronal activity evoked by the γ-aminobutyric acid A (GABA_A_) receptor antagonist stimulated SNCA secretion into the interstitial fluid of freely-moving mice. These findings indicate that SNCA is physiologically secreted to the extracellular space, and the SNCA secretion is stimulated by neuronal activity. We hypothesized that neuronal activity acted as a trigger to drive secretory autophagy for releasing SNCA extracellularly in neurons.

**Figure 1.**
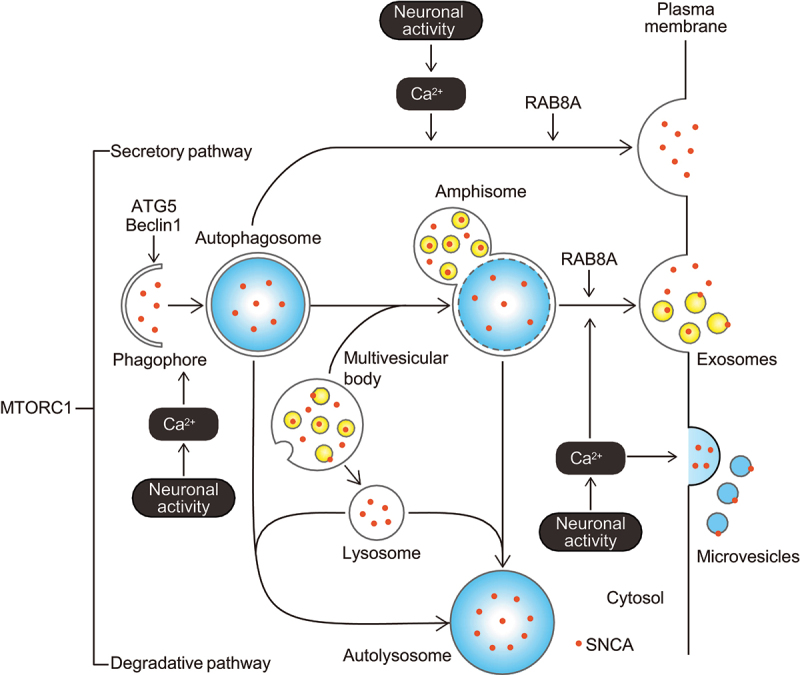
Hypothetical model of autophagic SNCA/α-synuclein secretion induced by neuronal activity. Stimulating neuronal activity promotes autophagic secretion of SNCA via an increase in intracellular Ca^2+^ concentration, and autophagosomes/amphisomes act as carriers for SNCA extracellular secretion.

To test this hypothesis, we investigated changes in autophagic flux and SNCA secretion by treating mouse primary cortical neurons with chemical agents and by transfecting undifferentiated SH-SY5Y cells stably expressing SNCA with cDNA or siRNAs [1]. In primary neurons, glutamate-induced hyperactivity or highly concentrated KCl-induced depolarization promoted SNCA secretion. Stimulating neuronal activity by glutamate promoted SNCA secretion in a manner dependent on an increase in intracellular Ca^2+^ concentration. The MTORC1 (mechanistic target of rapamycin kinase complex 1) inhibitor rapamycin also promoted SNCA secretion, while the effect of rapamycin on SNCA secretion was inhibited in primary neurons from *Becn1* (beclin-1) deficient (*Becn1*^+/-^) mice. Additionally, the promoting effect of glutamate on SNCA secretion was significantly suppressed by *Atg5* knockdown in SH-SY5Y cells. Moreover, glutamate-induced neuronal activity promoted autophagic flux and increased the colocalization of SNCA with LC3, a marker of autophagosomes, in primary neurons. All these effects of glutamate were inhibited by BAPTA-AM, the membrane-permeant Ca^2+^ chelator. Glutamate-induced neuronal activity did not affect the extent of colocalization of SNCA with LAMP1 (lysosomal-associated membrane protein 1)-positive structures. In fractionation experiments of conditioned media from primary neurons, endogenous SNCA was detected broadly in microvesicle-rich large extracellular vesicle, exosome-rich small extracellular vesicle, and free protein fractions, but it was found more abundantly in large and small extracellular vesicle fractions than in free protein fractions. Additionally, knockdown of *Rab8a*, which carries out fusion of autophagosomes and amphisomes with the plasma membrane, reduced neuronal activity-mediated SNCA secretion in SH-SY5Y cells. These findings show that neuronal activity triggers autophagic secretion for releasing SNCA with extracellular vesicles via an increase in intracellular Ca^2+^ concentration (Figure 1). Additionally, stimulating neuronal activity may divert the cargo from the degradative pathway towards the secretory pathway. In support of this scenario, neuronal activity is demonstrated to modulate synaptic plasticity via promotion of autophagic secretion in concert with SNAP29, SEC22 and RAB8, but diminishes autophagic degradation in *Drosophila*.

In this study, we also found that stimulating neuronal activity by glutamate promoted autophagic secretion of tau and SOD1 (superoxide dismutase 1), which are aggregate-prone proteins responsible for Alzheimer disease and a subgroup of familial amyotrophic lateral sclerosis, respectively [1]. These findings may reflect that neuronal activity-mediated secretory autophagy is a common pathway for clearance of aggregate-prone proteins associated with the pathogenesis of various neurodegenerative diseases. Future studies are needed to elucidate whether neuronal activity-induced secretory autophagy mediates SNCA aggregates and what factors regulate the switch between secretory and degradative pathways in autophagy.
